# An exploratory pilot study on the involvement of APOE, HFE, C9ORF72 variants and comorbidities in neurocognitive and physical performance in a group of HIV-infected people

**DOI:** 10.1007/s11011-022-00975-w

**Published:** 2022-03-30

**Authors:** Isabella Zanella, Eliana Zacchi, Chiara Fornari, Benedetta Fumarola, Melania Degli Antoni, Daniela Zizioli, Eugenia Quiros-Roldan, the Inflammation in H. I. V. Study Group

**Affiliations:** 1 Department of Molecular and Translational Medicine, University of Brescia, 25123 Brescia, Italy; 2 Clinical Chemistry Laboratory, Cytogenetics and Molecular Genetics Section, Diagnostic Department, ASST Spedali Civili Di Brescia, 25123 Brescia, Italy; 3 Department of Clinical and Experimental Sciences, University of Brescia, 25123 Brescia, Italy; 4 Division of Infectious and Tropical Diseases, ASST Spedali Civili Di Brescia, 25123 Brescia, Italy

**Keywords:** HIV, Neurocognitive/physical performance, Comorbidities, APOE, HFE, C9ORF72

## Abstract

Cognitive decline of aging is modulated by chronic inflammation and comorbidities. In people with HIV-infection (PWH) it may also be affected by HIV-induced inflammation, lifestyle and long-term effects of antiretroviral therapies (ART). The role of genetics in the susceptibility to HIV-associated neurocognitive disorders (HAND) is not fully understood. Here we explored the possible relations among variants in 3 genes involved in inflammation and neurodegenerative disorders (APOE: ε2/ε3/ε4; HFE: H63D; C9ORF72: hexanucleotide expansions ≥ 9 repeats), cognitive/functional impairment (MiniMental State Examination MMSE, Clock Drawing Test CDT, Short Physical Performance Battery SPPB), comorbidities and HIV-related variables in a cohort of > 50 years old PWH (n = 60) with at least 10 years efficient ART. Patients with diabetes or hypertension showed significantly lower MMSE (p = .031) or SPPB (p = .010) scores, respectively, while no relations between HIV-related variables and cognitive/functional scores were observed. Patients with at least one APOEε3 allele had higher CDT scores (p = .019), APOEε2/ε4 patients showing the lowest scores in all tests. Patients with HFE-H63D variant showed more frequently hypertriglyceridemia (p = .023) and those harboring C9ORF72 expansions > 9 repeats had higher CD4^+^-cell counts (p = .032) and CD4% (p = .041). Multiple linear regression analysis computed to verify possible associations among cognitive/functional scores and all variables further suggested positive association between higher CDT scores and the presence of at least one APOEε3 allele (2,2; 95% CI [0,03 0,8]; p = .037), independent of other variables, although the model did not reach the statistical significance (p = .14). These data suggest that in PWH on efficient ART cognitive abilities and physical performances may be partly associated with comorbidities and genetic background. However, further analyses are needed to establish whether they could be also dependent and influenced by comorbidities and genetic background.

**Supplementary Information:**

The online version contains supplementary material available at 10.1007/s11011-022-00975-w.

## Introduction

Cognitive decline is a hallmark of aging, but it can be modulated by multiple conditions, most of them related with chronic inflammation and more frequent in older people, including metabolic syndrome, elevated blood pressure, hyperglycemia and dyslipidemia. People with HIV infection (PWH) may be disproportionately impacted by cognitive decline through a variety of mechanisms such as HIV-induced inflammation, the long-term effects of antiretroviral therapy (ART), lifestyle (i.e., drug or alcohol use), psychiatric, and age-associated comorbidities (i.e., heart disease, hypertension, diabetes) probably accelerating and accentuating the characteristic premature aging among PWH (Waldrop et al. 2021; Aung et al. 2021).

Despite of efficient ART, the prevalence of neurocognitive deficits in PWH, collectively termed HIV-associated neurocognitive disorders (HAND), remains high and HIV-1 infection is associated with up to 40–50% of patients with HAND although, thanks to ART, HAND currently presents with milder symptoms (mild/moderate cognitive decline or MCD). The neuropathogenesis of HAND is now believed to be largely the result of neurocognitive and/or motor decline, driven by chronic brain inflammation (Hong and Banks 2015; Farhadian et al. 2017; Shinjyo and Kita 2021; Borrajo et al. 2021). It is well-known that some HIV proteins can activate macrophages, astrocytes and microglial cells in the brain, leading to the production of inflammatory molecules and damaging neurons, in turn resulting in neurocognitive impairment (Kaul et al. 2001; Ramesh et al. 2013).

Some human genotypes have been associated, through different mechanisms, to chronic neuroinflammation and consequently with deficits in cognitive or motor performance (Villegas-Llerena et al. 2016; Bright et al. 2019). Chronic inflammation in the brain, characterized by increased activation of microglia, is indeed a common feature of several neurodegenerative diseases, like amyotrophic lateral sclerosis (ALS), frontotemporal dementia (FTD), Parkinson’s disease (PD) and Alzheimer’s disease (AD), known to be at least in part genetically determined disorders (DiSabato et al. 2016; Yang and Zhou 2019).

The role of host genetics in the susceptibility to HAND is still not fully understood, although some variants may increase the risk to develop HAND (Olivier et al. 2018).

The chromosome 9 open reading frame 72 gene (C9ORF72) has been indicated in many cellular processes, including vesicle trafficking, lysosome homeostasis, mammalian target of rapamycin complex I (mTORC1) signaling and autophagy. C9ORF72 also affects inflammation, regulating the trafficking and lysosomal degradation of inflammatory mediators, including toll-like receptors (TLRs) and the signaling protein stimulator of interferon genes (STING) (Pang and Hu 2021), recently indicated as an important regulator in neurological infections, neuroimmunological diseases and neurodegenerative disorders Chin 2019). A large expansion of a GGGGCC hexanucleotide (hundreds or thousands of repeat units) within the non-coding region of the C9ORF72 gene is the main genetic cause of ALS and FTD (DeJesus-Hernandez et al. 2011), both characterized by the aggregation of misfolded proteins that promotes surrounding glia and peripherally derived immune cells, initiating a non-cell autonomous inflammatory process. Haploinsufficiency of C9ORF72 has also been implicated in abnormal microglia activation in those neurodegenerative disorders, given its roles in the inflammatory pathways above cited (Pang and Hu 2021). While the general population commonly harbor alleles with less than 30 repeats (mostly with 2, 5 or 8 repeats), C9ORF72 intermediate hexanucleotide expansions (9 to 30 repeat units) are more frequent in neurodegenerative and psychiatric diseases (Ng and Tan 2017; Cali et al. 2019; Kobo et al. 2021; Serpente et al. 2021) and have been recently also associated with autoimmune and severe infectious diseases (Tiloca et al. 2018; Fredi et al. 2019; Biasiotto and Zanella 2019; Zanella et al. 2021a).

The homeostatic iron regulator HFE gene, one of the main genes involved in human hereditary hemochromatosis (HH), codes for a major histocompatibility complex (MHC) class I protein, implicated both in iron homeostasis and immunity (Reuben et al. 2017). HH is mainly associated with homozygosity for the p.Cys282Tyr (C282Y) variant, while compound heterozygotes with the C282Y and p.His63Asp (H63D) variants have lower risk of developing HH and complications of iron overload and H63D homozygotes are considered at very low risk of HH. Although the H63D variant is less studied than the C282Y one (much rarer than the H63D variant) regarding the implications on immune functions, it also seems to have an impact on inflammatory and immune responses (Zanella et al. 2021b). H63D variant has also been repeatedly suggested to be a risk factor or a genetic modifier in neurodegenerative diseases like ALS, AD, PD and FTD, possibly due to its involvement in oxidative-stress, endoplasmic reticulum stress, autophagy and lipid metabolism in the brain (Gazzina et al. 2016; Kim and Connor 2020).

Apolipoprotein E (APOE) gene, primarily expressed by astrocytes and activated microglia in the brain, is a major responsible for lipid and cholesterol traffic, affecting various normal cellular processes, including neuronal growth, repair and remodeling of membranes, synaptogenesis, clearance and degradation of amyloid β (Aβ) and neuroinflammation (Shi and Holtzman 2018). In humans, the APOE gene has three common allelic variants, termed ε2, ε3 and ε4. APOE ε3/ε3 is the most common genotype and the APOE ε2/ε2 the least frequent. The APOEε4 allele is considered the strongest genetic risk factor for AD, whereas the APOEε2 allele is reported as neuroprotective in this disorder. Despite several reports that PWH harboring the APOEε4 allele have increased susceptibility to reduced cognitive functions, the role of this allele in HAND is still controversial (Olivier et al. 2018; Geffin and McCarthy 2018).

Therefore, HAND may be driven by several mechanisms, HIV-induced inflammation probably being the main driving factor, while ART, comorbidities, life-style and genetic background being possible exacerbating determinants. While genetic susceptibility is a non-modifiable risk factor for cognitive impairment, modifiable risk factors provide an opportunity for intervention and prevention of cognitive ability alterations and they have been studied in both HIV-infected and uninfected individuals (Foley et al. 2010; Fogel et al. 2015; Callisaya et al. 2019; Sun et al. 2020; Buyo et al. 2020; Xu et al. 2021; Tahmi et al. 2021).

In this study, we aimed to explore the prevalence of three possible genetic causes of neuroinflammation (C9ORF72 hexanucleotide repeat expansions above 8 units, H63D variant in the HFE gene and APOE ε2, ε3 and ε4 alleles) and their possible relation with cognitive/physical function impairment, comorbidities and HIV-related variables in a group of > 50 years old HIV-infected patients with at least 10 years of efficient ART.

## Materials and methods

### Patients

This exploratory study was conducted from November 2019 to February 2020 at ASST Spedali Civili Hospital, Brescia, Italy. The recruitment for the study was stopped in February 2020 because of COVID-19 pandemic. We enrolled all patients attending to their HIV visit that had the following inclusion criteria: aged > 50 years old, at least 10 years of ART that gave the written consent to participate. Patients were excluded if they had psychiatric disease (including depression), assumed anticholinergic drugs, declared high alcohol intake or psychotropic drugs use or had previous or current opportunistic infections in the brain.

The study involved a blood sample for the genetic analysis and the neurocognitive/physical function assessment. HIV-related values and comorbidities were collected from the medical chart of each patient.

The study was approved by the Ethics Committee of University of Brescia and was conducted in accordance with the Declaration of Helsinki (2013) and with the principles of Good Clinical Practice. Written informed consent to participate in the study was obtained.

### Genetic analysis

Genomic DNA was extracted from peripheral blood using the Wizard Genomic DNA Purification kit (Promega), according to the manufacturer’s instructions. C9ORF72 hexanucleotide expansion size was determined with a polymerase chain reaction (PCR)-based two-step analysis, as previously described (Biasiotto et al. 2017). A cut-off of ≥ 9 repeat units was chosen to distinguish short (2–8) from intermediate (9–30) or large (> 30) hexanucleotide expansions, on the basis of previously described criteria (Fredi et al. 2019). HFE genotyping was determined by PCR amplification and direct Sanger sequencing, with specific primers (Gazzina et al. 2016). APOE genotyping was determined by PCR amplification and direct Sanger sequencing, with specific primers (forward primer: 5’- GGCACGGCTGTCCAAGGA reverse primer: 5’- CAGGCGCTCGCGGATG). Sanger sequencing was performed on the ABI 3500 Genetic Analyzer (ThermoFisher Scientific).

### Neurocognitive and physical function assessment

Patients underwent a session of neurocognitive and physical function assessment in which three tests were administered to evaluate their cognitive and physical abilities. The Italian standardization (Magni et al. 1996) of the Mini-Mental State Examination (MMSE) (Folstein et al. 1975) is composed of 30 items that assess different cognitive functions, such as attention, orientation, memory, calculation, language and visual spatial skills (Arevalo-Rodriguez et al. 2015). MMSE is a widely used test in clinical practice to assess cognitive impairment (Magni et al. 2016; Quintino-Santos et al. 2015; Arevalo-Rodriguez et al. 2015). MMSE scores range from zero to 30 and are corrected for age and years of schooling in accordance with the Italian validation (Magni et al. 1996). A cut-off of 24 was used to detect the presence of cognitive impairment. Mild/moderate cognitive decline (MCD) was considered with MMSE score between 11 and 24 and severe cognitive decline (SCD) when MMSE was < 10 (Perneczky et al. 2006).

The Clock Drawing Test (CDT) (Sunderland et al. 1989) is a simple measure of visuo-spatial, planning, abstraction abilities and executive functions in general (Shulman 2000; Mainland and Shulman 2017). CDT scores range from 1 to 10. A cut-off of 5 was used to detect the presence of cognitive impairment (Sunderland et al. 1989).

The Short Physical Performance Battery (SPPB) (Guralnik et al. 1995) allows to quickly assess the physical functions in older adults. The SPPB is composed of 3 parts: the balance test, the gain speed test and the chair stand test. SPPB scores range from 0 to 12. The score is divided in classes of limitations: 0–3 points mean severe limitations; 4–6 moderate limitations; 7–9 mild limitations; 10–12 minimal limitations (Guralnik et al. 1995).

### Statistical analysis

Continuous variables were reported as median and interquartile range (Me ± IQR) and were compared using the nonparametric Kruskal–Wallis test. Categorical variables were summarized through frequency and percentages and were compared using the Fisher’s Exact test (for 2 × 2 labels with frequencies lower than 5) and the Chi-Squared test. Furthermore, Spearman correlations were computed between continuous variables. Moreover, we analysed the possible differences between the presence of multiple gene mutations and both the medical history and the cognitive performance using the Fisher’s exact tests and the Kruskal–Wallis test, respectively. Lastly, three multiple regressions were computed for cognitive/function performances scores (CDT, MMSE and SPPB, as dependent variables), using the Enter data method to investigate their association with the genetic variants (expansion of the hexanucleotide repeat in the C9ORF72 gene above 8 units; APOEε2, APOEε3, APOEε4 alleles; H63D variant in the HFE gene), clinical history (hypertriglyceridemia, diabetes, hypercholesterolaemia, cardiovascular disease, hypertension, liver disease, kidney disease, cancer and BMI), demographic data (age) and with the HIV-related characteristics (CD4^+^ T cells count, nadir of CD4^+^ T cells count, CD8^+^ T cells count, zenith of CD8^+^ T cells count, years on ART, previous virologic failure to ART, plasmatic HIV RNA). All the continuous variables were transformed into logarithms (log10) because the Shapiro–Wilk test was violated. Statistical significance level was set at α = 0.05 and 95% CI were computed. Statistical analysis was performed with JASP (version 0.12.2.0).

## Results

### Demographic and clinical characteristics

We consecutively recruited 60 HIV-infected patients. A total of 45 males (75%) and 15 females (25%) participated to the study (median age ± IQR: 69,5 ± 13,8 years; median years of schooling ± IQR: 8 ± 8; median BMI ± IQR: 24,7 ± 5; median years on ART ± IQR: 18 ± 13), most patients were non-smokers (60.7% of patients) (Supplementary Table S1). Hypertriglyceridemia (38,3%), hypercholesterolemia (35%), liver disease (33,3%), hypertension (31,7%) and cardiovascular disease (26,7%) were the most common comorbidities (Table 1). More than half of patients had at least two comorbidities (58,3%) and only in 15% of patients (n = 9) no comorbidities were identified. Respect to HIV-related values, median of CD4^+^ T cell count (± IQR) was 546/ mm^3^ ± 356,5, median nadir of CD4^+^ T cell count was 136/mm^3^ ± 224,5 and median of CD8^+^ T cell count was 672,5/mm^3^ ± 577,3. Most patients (91, 1%) had plasmatic HIV RNA < 20 copies/ml and had no previous virologic failure (73,3% of patients) (Supplemental Table S1).

**Table 1:** Prevalence of comorbidities

Comorbidities	Frequency (%) (N = 60)	Comorbidities	Frequency (%) (N = 60)
Hypertriglyceridemia		Hypertension	
No	37 (61,7%)	No	41 (68,3%)
Yes	23 (38,3%)	Yes	19 (31,7%)
Diabetes		Liver disease	
No	50 (83,3%)	No	40 (66,7%)
Yes	10 (16,7%)	Yes	20 (33,3%)
Hypercholesterolaemia		Kidney disease	
No	39 (65%)	No	47 (78,3%)
Yes	21 (35%)	Yes	13 (21,7%)
Cardiovascular disease		Cancer	
No	44 (73,3%)	No	50 (83,3%)
Yes	16 (26,7%)	Yes	10 (16,7%)

Hypertriglyceridemia was considered when patients were taking omega-3 fatty acids and/or fibrates or persistently showed triglyceridemia > 150 mg/dL. Diabetes was considered when patients were taking antidiabetic drugs or persistently showed glycemia > 120 mg/dL. Hypercholesterolemia was considered when patients were taking statins or persistently showed cholesterolemia > 200 mg/dL. Cardiovascular diseases were defined if patients had history of coronary heart disease, cerebrovascular disease, peripheral arterial disease, rheumatic heart disease, congenital heart disease, aortic disease and/or deep vein thrombosis and pulmonary embolism. Hypertension was considered when patients were taking anti-hypertensive drugs. Liver disease was considered if patients were Hepatitis C virus (HCV) ab or Hepatitis B s Antigen (HBsAg) positive. Kidney diseases was considered when clearance of creatinine < 80 ml/min. Cancer was considered when patients have current or had previous history of cancer

### Neurocognitive and physical function assessment

A total of 6 patients and 5 patients refused to execute the neurocognitive and functional assessments, respectively, after giving the consent and blood sampling.

Median scores of MMSE (26,2 ± 2,3), CDT (9 ± 5) and SPPB (11 ± 3) were normal (without statistical differences between males and females). MMSE was altered in 9 (16,7%) patients (score < 25; Me = 19,9 ± 6), among them 8 patients with MCD (score 11–24, Me = 21 ± 5) and only one with SCI (score < 10; MMSE scores = 8,9). CDT was altered in 15 (27,8%) patients, most of them (14 patients) scored between 4–5 and only one scored 1. MMSE and CDT were both pathological in 5 (9,3%) of patients. At last, SPPB was altered (with at least mild limitations) in 18 (32,7%) patients (Table2).

**Table 2:** Neurocognitive and physical function assessment

Characteristics				p-values*
MMSE. Frequency (%)				.418
	Male (N = 40)	Female (N = 14)	Total (N = 54)	
Normal	32 (80%)	13 (92,9%)	45 (83,3%)	
Pathological	8 (20%)	1 (7,1%)	9 (16,7%)	
N-miss	5	1	6	
MMSE score. Median (IQR)				
	Male (N = 40)	Female (N = 14)	Total (N = 54)	
Total score	26,1 (2,1)	27 (3,1)	26,2 (2,3)	.221^$^
	Male (N = 32)	Female (N = 13)	Total (N = 45)	
Normal score	27 (2,1)	27 (3,4)	27 (2,1)	
	Male (N = 8)	Female (N = 1)	Total (N = 9)	
Pathological score	21 (6)	24 (0)	19,9 (6)	
Mild/moderate (N = 8)	22 (5,3)	24 (0)	21 (5)	
Severe (N = 1)	8,9 (0)	-	8,9 (0)	
CDT. Frequency (%)				1.000
	Male (N = 40)	Female (N = 14)	Total (N = 54)	
Normal	29 (72,5%)	10 (71,4%)	39 (72,2%)	
Pathological	11 (27,5%)	4 (28,6%)	15 (27,8%)	
N-miss	5	1	6	
CDT score. Median (IQR)				.562
	Male (N = 40)	Female (N = 14)	Total (N = 54)	
Total score	9 (5)	8 (4,5)	9 (5)	
	Male (N = 29)	Female (N = 10)	Total (N = 39)	
Normal score	10 (2)	8,5 (2)	10 (2)	
	Male (N = 11)	Female (N = 4)	Total (N = 15)	
Pathological score	5 (1)	4 (1)	5 (1)	
MMSE and CDT. Frequency (%)				.426
	Male (N = 40)	Female (N = 14)	Total (N = 54)	
MMSE and CDT pathological	5 (12,5%)	0 (0%)	5 (9,3%)	
MMSE pathological, CDT normal	3 (7,5%)	1 (7,1%)	4 (7,4%)	
MMSE normal, CDT pathological	6 (15%)	4 (28,6%)	10 (18,5%)	
MMSE and CDT normal	26 (65%)	9 (64,3%)	35 (64,8%)	
SPPB. Frequency (%)				.497
	Male (N = 41)	Female (N = 14)	Total (N = 55)	
Minimal limitations	29 (70,7%)	8 (57,1%)	37 (67,3%)	
Mild limitations	9 (22%)	5 (35,7%)	14 (25,5%)	
Moderate limitations	2 (4,8%)	0 (0%)	2 (3,6%)	
Severe limitations	1 (2,4%)	1 (7,1%)	2 (3,6%)	
N-miss	4	1	5	
SPPB score. Median (IQR)				.688
	Male (N = 41)	Female (N = 14)	Total (N = 55)	
Total score	11 (3)	10,5 (4)	11 (3)	

Neurocognitive tests were corrected in accordance with the Italian validations (see methods). MMSE (Mini Mental State Examination). Normal: MMSE score > 24. Mild-moderate cognitive impairment: 11 ≤ MMSE score ≥ 24. Severe cognitive impairment: MMSE score < 10. CDT (Clock Drawing Test). *p values were calculated by Kruskal–Wallis test, Fisher’s exact test and Chi-Squared test, as appropriate. ^$^The p-value was computed for the total score because observations < 2 occur in the pathological MMSE score

No statistically differences in the neurocognitive/functional tests’ score linked to the presence/absence of comorbidities were found except that MMSE score was lower in patients with diabetes than those who did not have diabetes (median 25,2 vs 26,3; p = 0.031) and SSPB score was lower in patients with arterial hypertension respect to those without (median 9 vs 11,5; p = 0.010). Moreover, significant negative correlations were found between age and CDT score (Spearman’s rho = -0.27, 95% CI [-0.002 -0.501]; p = 0.049) and age and SPPB score (Spearman’s rho = -0.338, 95% CI [-0.08 -0.554]; p = 0.012), while no statistically significant correlation between cognitive/functional test scores and HIV characteristics were found including CD4^+^ T cell count and years on ART (Supplemental Tables S2 and S3).

### Genetic analyses

Regarding the hexanucleotide expansion in the C9ORF72 gene, 8 patients (13,3%) had at least one allele with an expansion ≥ 9 hexanucleotide repeat units, of which one harbored both an intermediate (≥ 9 but < 30 repeats) and a large (> 30 repeats) expansion. Regarding the APOE gene alleles, most of the participants (96,7%) harbored at least one ε3 allele (26,7% of patients with a single ε3 allele vs 70% of patients with a double ε3 allele); 10 patients (16,7%) were heterozygous for the ε2 allele (no ε2/ε2 homozygous, 8 compound ε2/ε3 heterozygous and 2 compound ε2/ε4 heterozygous subjects), while 10 patients (16,7%) had a single ε4 allele (no ε4/ε4 homozygous, 2 compound ε2/ε4 heterozygous and 8 compound ε3/ε4 heterozygous subjects). Considering the H63D variant in the HFE gene, 28,3% of patients were heterozygous for the variant and only one patient (1,7%) had a double H63D allele (Table 3 and Supplemental Table S4). Finally, 6 patients harbored more than one variant: 2 participants (3,3%) had an intermediate expansion in the C9ORF72 gene and a single ε4 allele in the APOE gene; 2 patients (3,3%) had the H63D variant in the HFE gene and a single ε4 allele in the APOE gene; one patient (1,7%) had an intermediate expansion in the C9ORF72 gene and the HFE H63D variant; one patient (1,7%) harbored three variants, a C9ORF72 intermediate expansion, the HFE H63D variant and one APOEε4 allele.

**Table 3:** Prevalence of variants

Characteristics	Frequency (%)			p-value
	Male (N = 45)	Female (N = 15)	Total (N = 60)	
C9ORF72 repeats ≥ 9				.400
No	40 (77,9%)	12 (80%)	52 (86,7%)	
Yes	5 (11,1%)	3 (20%)	8 (13,3%)	
APOEε2 (n° alleles)				1.000
0	37 (82,2%)	13 (86,7%)	50 (83,3%)	
1	8 (17,8%)	2 (13,3%)	10 (16,7%)	
2	0 (0%)	0 (0%)	0 (0%)	
APOEε3 (n° alleles)				.073
0	1 (2,2%)	0 (6,7%)	2 (3,3%)	
1	9 (20%)	7 (46,7%)	16 (26,7%)	
2	35 (77,8%)	7 (46,7%)	42 (70%)	
APOEε4 (n° alleles)				.001*
0	42 (93,3%)	8 (53,3%)	50 (83,3%)	
1	3 (6,7%)	7 (46,7%)	10 (16,7%)	
2	0 (0%)	0 (0%)	0 (0%)	
HFE H63D (n° alleles)				.177
0	33 (73,3%)	9 (60%)	42 (70%)	
1	12 (26,7%)	5 (33,3%)	17 (28,3%)	
2	0 (0%)	1 (6,7%)	1 (1,7%)	

^*^ p < .05. Fisher’s exact test and Chi-squared test were applied as appropriate

For further analyses, we grouped the only one homozygous patient for the HFE H63D variant with patients harboring a single H63D allele and the only one patient harboring both an intermediate and a large C9ORF72 expansion together with the other patients with intermediate expansions.

**Fig. 1:**
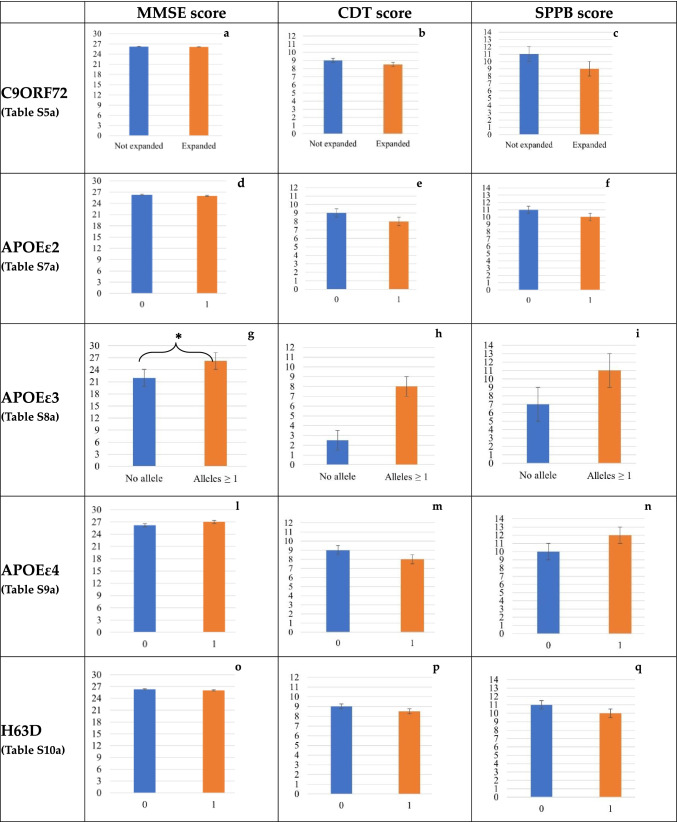
Neurocognitive/functional assessment and genetics. The figure represents the differences in the neurocognitive/functional tests’ scores (MMSE, CDT and SPPB) linked to the C9ORF72 (a, b, c) expansions, to the APOEε2 (d, e, f), APOEε3 (g, h, i) and APOEε4 (l, m, n) alleles, to the HFE H63D (o, p, q) variants. X axes represent the number of alleles or if the variant is present or not (e.g. C9ORF72, APOEε3). Y axes represent the median score at the cognitive/functional assessments. Vertical bars represent standard errors. * marks significant values

We analyzed the differences in the neurocognitive/functional tests’ scores relatively to the presence/absence of each genetic variant (Supplemental Tables S5a-S10a). We only identified a significantly higher CDT score for subjects harboring the APOEε3 variant in at least one allele compared with those with no ε3 alleles (median score 8 vs 2,5; p = 0.019), that corresponded to the 2 subjects with the APOE ε2/ε4 genotype showing the lowest scores in all the tests. Patients carrying the APOE ε2/ε4 also performed worse in MMSE and SPPB tests although the difference did not reach the statistical significance (Fig. 1 and Supplemental Table S6a).

Regarding the differences in the presence/absence of comorbidities relatively to the presence/absence of each genetic variant, we only found a significantly higher proportion of patients with hypertriglyceridemia in patients with the H63D variant in the HFE gene (p = 0.023) (Supplemental Table S10b). Although few cases, we found that none of the subjects harboring the APOε4 had current or history of cardiovascular disease (p = 0.05) (Supplemental Table S9b).

We also explored the differences between HIV-related characteristics and presence/absence of each genetic variant. We found significant differences in CD4^+^ T cell count relatively to the C9ORF72 hexanucleotide expansion, in particular those patients who harbored an expansion ≥ 9 repeat units had also higher CD4^+^ T cell count and CD4% than those with shorter expansions (median CD4^+^ T cell count 776/mm^3^ vs 513,5; p = 0.032; median CD4% 38,6 vs 28,5; p = 0.041) (Supplemental Tables S5c and S6c).

Finally, we explored the differences among patients who harbored variants in more than one among the studied genes (n = 6), and comorbidities or neurocognitive/functional test’ scores. No significant differences were found between those who had one variant and those who presented with more than one variant (data not shown).

### Multiple linear regression analyses

We computed three linear multiple regressions one for each dependent variable (MMSE, CDT and SPPB) to analyze the association among neurocognitive/functional scores with genetic variables, HIV-related characteristics (CD4^+^ T cell count and percentage, nadir of CD4^+^ T cell count, CD8^+^ T cell count and zenith of CD8^+^ T cell count, years on ART and previous virologic failures to ART), clinical history (hypertriglyceridemia, diabetes, hypercholesterolemia, cardiovascular disease, hypertension, liver disease, kidney disease, cancer and BMI) and demographic data (age). Only the model built on CDT score showed a trend to significance (F (22, 31) = 1,52; p = 0.14), suggesting that CDT score could be positively associated with the APOEε3 allele (2,2; 95% CI [0,03 0,8]; p = 0.037). Multiple regressions computed to ascertain the associations among MMSE and SPPB scores with variables were not significant (ps > 0.05) (Table 4 and Supplemental Table S11).

**Table 4:** Coefficients from the multiple regression of CDT score

							95% CI		Collinearity Statistics	
Model		Unstandardized	Standard Error	Standardized	t	p	Lower	Upper	Tolerance	VIF
H_0_	(Intercept)	861	25		33,982	< .001	810	912		
H_1_	(Intercept)	531	737		721	477	-972	2035		
	C9ORF72 (alleles ≥ 9)	76	120	108	632	532	-169	321	532	1879
	APOEε2	-37	93	-74	-391	699	-227	154	435	2299
	APOEε3	435	199	445	2182	37	28	841	373	2679
	APOEε4	-18	106	-37	-173	863	-236	199	336	2980
	HFE H63D	-45	71	-111	-631	533	-189	100	505	1982
	CD4 + T cells count	111	190	146	586	562	-277	499	251	3986
	Nadir CD4	-54	75	-151	-713	481	-208	100	346	2893
	CD8 + T cells count	-77	169	-113	-454	653	-423	269	251	3983
	Zenith CD8	76	178	99	426	673	-287	439	290	3445
	Age	-5	4	-289	-1297	204	-14	3	313	3194
	Years on ART	2	4	83	481	634	-6	10	521	1919
	Previous virological failure to ART	5	32	30	170	866	-60	71	500	1999
	Plasmatic HIV RNA	25	111	35	224	824	-202	251	625	1599
	BMI	-1	6	-45	-214	832	-13	10	353	2833
	Hypertriglyceridemia	111	94	288	1191	243	-79	302	265	3776
	Diabetes	-8	93	-17	-91	928	-198	181	442	2261
	Hypercholesterolaemia	-14	98	-35	-140	890	-214	187	254	3943
	Cardiovascular disease	51	65	122	784	439	-82	185	644	1554
	Hypertension	39	78	100	499	621	-120	198	391	2558
	Liver disease	-15	68	-38	-216	830	-152	123	507	1971
	Kidney disease	9	90	18	95	925	-176	193	430	2328
	Cancer	-12	76	-26	-164	871	-167	142	610	1640

Multiple regression of CDT score as dependent variable (F (22, 31) = 1,52; p = .14)

## Discussion

Modern ART has greatly improved the lives of PWH, although they are aging with a high burden of chronic inflammatory-related diseases, such as heart diseases, diabetes or cancer and cognitive impairment. Here we explored if cognitive/functional performance could also be influenced by genetic background together with HIV infection or comorbidities.

In this group of PWH aged > 50 years and with more than 10 years of efficient ART, a high burden of comorbidities (58,3% of patients with more than one comorbidity) and with a prevalence of the analyzed variants in 3 genes, possibly linked to neuroinflammation, similar to the general population, cognitive impairment was present in 35,2% of patients (pathologic MMSE and/or CDT), all of them with MCD but one with SCI. Cognitive impairment (lower MMSE test score) was more frequent in patients with diabetes. SPPB was pathologic in 32,7% of patients and physical limitations, although mild, were more frequent in patients with hypertension. No HIV-related variable was significantly associated in our study with neurocognitive/functional impairment except for CD4^+^ T cells and C9ORF72 hexanucleotide expansion. The absence of the APOEε2 and APOEε4 alleles in the genetic background seems to be associated with better cognitive performance.

Mild Cognitive impairment (MCI) is the abnormality of cognitive functions in populations matched for age and education levels, but without loss of basic skills in everyday social and occupational life (here we did not evaluate these abilities). MCI is characterized by difficulties in remembering events, along with problems in orientation, planning, decision making, and instruction interpreting (Zhuang et al. 2021) and it is associated with a significant risk of developing dementia, but it does not always proceed to dementia, with approximately 18% of MCI reversing to normal cognition spontaneously in general population (Canevelli et al. 2016).

MMSE is a widely used cognitive assessment tool to detect global cognitive dysfunctions (orientation, memory, attention, language and calculation). It has high specificity (> 80%) with lower sensitivity (60%) to detect MCI (Zhuang et al. 2021). CDT is extensively used as cognitive screening test due to its simplicity and brevity, it assesses mainly prefrontal and parieto-occipital functions (processing speed, visuo-spatial, planning and abstraction abilities) (Shulman 2000; Mainland and Shulman 2017) and it has a specificity of 80% and a sensitivity of 77% for detecting cognitive impairment with a cut-off point of 5/6 (Carnero-Pardo et al. 2022). In the present study we tested only cognitive decline (MMSE and CDT) and not its impact in daily occupational activity, therefore MCD found in our study does not exactly correspond with the clinical diagnosis of MCI.

Prevalence of neurocognitive disorders in PWH varies widely depending on population studies (age, socioeconomic status, HIV stage, comorbidities) and the used diagnostic approach, reaching in some cohorts up to 40–50% (Wei et al. 2020). Currently, while severe cognitive decline in PWH is rare (2%), mild cognitive disorders are diagnosed in around 30–36% of patients (Wei et al. 2020). Cognitive decline was present in 44,4% of our patients (9 and 15 patients with pathological MMSE at CDT scores, respectively) only one of them (1,9%) with a severe deficit.

Similarly, data on the prevalence of diabetes in PWH is highly varied but the risk of developing diabetes in these patients can be four times greater compared to those without HIV. Diabetes can occur at an earlier age and even without the presence of obesity compared to those without HIV and it correlates with duration of HIV infection and consequently the duration of ART (Brown et al. 2005; Hernandez-Romieu et al. 2017; Tiozzo et al. 2021). In our study only 17% of patients had diabetes. It is known that people with diabetes, especially with poor glycemic control, are more vulnerable to cognitive dysfunction and diabetes is associated with reduced cognitive function (Callisaya et al. 2019; Sun et al. 2020; Xu et al. 2021). We indeed found that HIV-infected subjects with diabetes in our cohort had a lower MMSE score, confirming the above observations. In general, all vascular risk factors (such as hypertension, dyslipidemia, diabetes, heart disease and obesity) contribute to cognitive impairment in HIV-uninfected population although this association is less studied in PWH (Foley et al. 2010; Qin et al. 2021; McIntosh et al. 2021). Anyway, in PWH, an adequate pharmacological treatment for cardiovascular risk (including diabetes) treatment seems to ameliorate the cognitive impact of the risk (Foley et al. 2010).

SPPB measures physical or functional performance in older adults and it has been demonstrated useful also in PWH. Physical functional impairment was present in 32,7% of our patients, a prevalence similar to other studies with similar patients (low/moderate cardiovascular risk but 20 years younger) (Umbleja et al. 2020). We did not find an association for SPPB score with any of the HIV-related variables or genetic background. Only patients with hypertension had a lower SPPB score (p < 0.01), as already described (Umbleja et al. 2020), but no association with neurocognitive impairment was found.

Some genetic profiles have been associated also with cognitive functions in the general population. The relation between the APOEε4 variant and neurocognitive decline is discussed but it can be modulated with a healthy lifestyle (Jin et al. 2021; Perez-Lasierra et al. 2021).

As in previous studies (Quintino-Santos et al. 2015), we found no significant effect of the APOEε4 variant on the global score of MMSE. However, we found that the CDT score was linked to the APOEε3 allele and therefore the absence of the APOEε4 and/or APOEε2 variants. In detail, those subjects who harbored at least one APOEε3 allele had also a better performance in the executive function assessment, while patients harboring the APOEε4 variant had worse scores, although the difference did not reach the statistical significance (p = 0.068). This result highlights new cognitive outcomes linked to the APOE polymorphisms in PWH. Recently, lower CDT scores have been correlated with hypoperfusion in cortical and subcortical areas (Duro et al. 2019) and the correlation between some APOE variants and lipids and, therefore, microcirculation, is known. In PWH mild alterations in the CDT could be an early signal of cerebrovascular damage, especially in patients carrying APOE variant correlated with dyslipidemia. It has been demonstrated the cognitive benefit of statin use in these patients (de Oliveira et al. 2020).

In our cohort we found that the APOEε2ε4 subjects had worse performances in all tests, even lower than subjects with the ε3ε4 genotype, although the differences did not reach statistical significance (Fig. 1 and Supplementary Table S6a). The role of the ε2ε4 genotype in the development of cognitive decline is still controversial, also due to the small number of subjects harboring this rare genotype. Two longitudinal studies on large populations of old adults have recently concluded that the ε2ε4 carriers had a higher MCI risk than ε3ε3 carriers, suggesting that the toxic effect of the ε4 allele may prevail over the protective effect of the ε2 allele (Oveisgharan et al. 2018; Ren et al. 2020). This effect may explain our findings. Although we are conscious of the low number of subjects considered in our study, we may speculate that the ε2ε4 genotype, at least in the inflammatory context of HIV infection, could reflect in a more pronounced neuroinflammation leading to enhanced cognitive decline. In this respect, it is interesting to note that both the ε2 and the ε4 allele enhance the inflammatory response in myeloid cells through distinct pro-inflammatory mechanisms that are independent of their function in plasma lipoprotein transport (Igel et al. 2021).

In PWH the correlation between the APOEε4 allele and cognitive decline seems to be influenced by CD4^+^ cell count nadir (Yang et al. 2021). Here, we did not explore this hypothesis because of the scarce number of APOEε4 carriers; however, we did not find significant correlations between CD4^+^ T cell count nadir and cognitive scores, in contrast to previous observations (Valcour et al. 2006; Ellis et al. 2011).

Regarding the H63D variant in the HFE gene, in our cohort we found 28,3% heterozygotes and 1,7% homozygotes for the variant, frequencies that do not significantly differ from those described elsewhere in the Italian population (Hanson et al. 2001; Chiò et al. 2015). Although the H63D variant in the HFE gene has been thoroughly described as a risk factor or a genetic modifier in many neurodegenerative diseases (Kim and Connor 2020), in our cohort, we did not find any relationship between the presence of the variant and cognitive or physical abilities, accordingly to previous studies (Berlin et al.^2004^; Guerreiro et al. 2006; Blázquez et al. 2007; Tisato et al. 2018). We only found a relationship between the presence of the variant and hypertriglyceridemia in our cohort of HIV-infected subjects. This finding is not reported in the literature, although a link between the H67D variant (orthologous to the human H63D variant) in mice and altered cholesterol metabolism has been described, also in association with memory impairment (Ali-Rahmani et al. 2014). Hypertriglyceridemia has been linked with cerebrovascular pathology and neurodegeneration in murine models (Hoyk et al. 2018). Furtherly, interactions between the H63D variant and the APOEε4 allele as risk factors for the development of cognitive decline have been described in the literature, although we did not find any relationship in our cohort (Combarros et al. 2003; Pulliam et al. 2003; Percy et al. 2014). Then, considering the low number of patients with the H63D variant and the APOEε4 allele found in this pilot study, further longitudinal studies in larger cohorts of subjects harboring both variants are needed to elucidate the possible role in cognitive and functional impairment and their possible interactions, both in the general population and in HIV-infected subjects.

The C9ORF72 gene is particularly expressed in myeloid cells and its complete loss in C9orf72^−/−^ knock-out mice has been implicated in exaggerated inflammatory responses characterized by the activation of the type I interferon pathway (Pang and Hu2021). Human FTD and ALS patients with a C9ORF72 large hexanucleotide expansion show a neuroinflammation phenotype and an elevated type I interferon signature (McCauley et al. 2020). Interestingly, Herpes Simplex Virus 2 (HSV2) infection in the spinal cord of mice have been shown to decrease C9ORF72 expression, suggesting that this gene may also be modulated by viral infections of the central nervous system (Cabrera et al. 2020). C9ORF72 hexanucleotide expansions of intermediate length also seem to modulate C9ORF72 expression both in myeloid cells and the brain (Cali et al. 2019) and have been associated with PD, AD, corticobasal degeneration (CBD) and psychiatric symptoms (Ng and Tan 2017; Cali et al. 2019; Kobo et al. 2021; Serpente et al. 2021). The role of C9ORF72 intermediate repeats in MCI has not yet been thoroughly studied, but the only reports on this topic suggest that intermediate expansions do not associate with MCI (Cacace et al. 2013) or may associate with better cognition, measured by MMSE score (Kaivola et al. 2019). In our cohort of HIV patients, we did not find significantly worse cognitive or functional test scores in patients with intermediate expansions, except for a lower SPPB score, although these data did not reach the statistical significance, also due to the small number of subjects who performed the neurocognitive and physical function assessment (5 of 10 refused the tests). Surprisingly, the only one subject with a large C9ORF72 expansion had normal test scores. This can be explained considering that this mutation is not fully penetrant. Penetrance is age-dependent with median age at symptom onset being 58 years and this patient was 72 years old, however for this genetic variant the age of symptom onset may vary from 40 to 90 years of age and some carriers do not show symptoms even at an age over 90 years (Murphy et al. 2017). Patients with an expansion ≥ 9 repeat units had however significantly higher CD4^+^ T cell count and CD4^+^ T cell % than those with shorter expansions in our cohort. Intermediate C9ORF72 expansions have been associated with autoimmune diseases and susceptibility to severe infectious diseases, in accordance with the role of this gene in immune responses (Tiloca et al. 2018; Fredi et al. 2019; Biasiotto and Zanella 2019; Zanella et al. 2021a). The role of C9ORF72 in immunity has been mostly studied in myeloid cells, however C9orf72^−/−^ knock-out murine models showed T cell activation Atanasio et al. 2016) and CD4^+^ T cell expansion (Burberry et al. 2016), suggestive of a possible influence of the modulation of C9ORF72 expression by intermediate expansions on this T cell population. This observation deserves further studies in the general population and in larger HIV cohorts.

Our work has some strengths, to our knowledge it was one of the first studies that compares genetic polymorphisms, comorbidities, HIV-related values, and neurocognitive outcomes in HIV-infected patients. Previously, several authors studied the possible correlation between genetic polymorphisms, comorbidities or HIV-related values and neurocognitive outcomes in PWH (Foley et al. 2010; Fogel et al. 2015; Monroe et al., 2017; Callisaya et al. 2019; Sun et al. 2020; Buyo et al. 2020; Levine et al., 2020; Ojeda-Juarez and Kaul, 2021; Xu et al. 2021; Tahmi et al. 2021). Notwithstanding, the combination of cognitive and physical functionality with genetic, comorbidities and HIV related values has never been assessed. Although our study has not a definitive conclusion, it adds relevant new data to previous research that still requires larger numbers of well characterized PLWH in order to address the potential link between HAND and genetic background, comorbidities or HIV-related values.

Certain limitations must be considered. First the small sample size reduced the statistical power; second, the associations that we found do not demonstrate causality and can be biased by confounding variables that we did not consider. Third, our study design lacked HIV-uninfected controls to understand if HIV infection can be an additive factor. Moreover, the interpretability of the regression models’ results is limited because the variables were transformed due to the normality violation and the models were not valid. Finally, cross-sectional design of the study with a small sample size may limit the reliability of the results.

In conclusion, ART and treatment of comorbidities seems to guarantee a fair level of functioning in the cognitive abilities and in the physical performances in PWH aged > 50. Higher CDT score was measured in patients carrying at least one APOε3 allele independent of other HIV- or comorbidities-related variables.

The average age of HIV-infected individuals in Europe is now over 50 years of age, raising concern about possible synergies between HIV and aging to potentially exacerbate neurocognitive impairment. Mild impairment remains the most frequent form and the diagnosis and treatment of cofactors for neurocognitive impairment, such as hypertension, diabetes, obesity, and dyslipidemia remain the most important factors.

Future similar studies on this topic, especially those of longitudinal nature, are necessary to evaluate the evolution of the neurocognitive score in our patients. Studies covering all stages of the HIV-1 infection that include other clinical, medical (type of ART, lipids values), virologic, and psychosocial variables and patients with more severe neurocognitive impairment, in addition to more extensive neurocognitive evaluation tests are needed.

## Supplementary Information

Below is the link to the electronic supplementary material.

Supplementary file1 (PDF 823 kb)

## Data Availability

All study data, including raw and analyzed data, and materials will be available from the corresponding author on reasonable request.
