# Supplementary material for: An exploratory pilot study on the involvement of APOE, HFE, C9ORF72 variants and comorbidities in neurocognitive and physical performance in a group of HIV-infected people
Source: Metab Brain Dis. 2022 Mar 30;37(5):1569–83. doi: 10.1007/s11011-022-00975-w (PMC8964929; doi:10.1007/s11011-022-00975-w)
Supplement: Supplementary file 1 — (PDF 823 kb) [file 11011_2022_975_MOESM1_ESM.pdf]

LIST of TABLES - SUPPLEMENTARY INFORMATION

Table	Title
	Patients characteristics (epidemiological, comorbidities, HIV)………...
	Neurocognitive/functional outcomes and comorbidities………………..
	Spearman's Correlations……………………………………………...
	Prevalence of APOE genotype……………………………………….
	C9ORF72……………………………………………………………….. (a) C9ORF72, neurocognitive/functional assessment<br>(b) C9ORF72 and comorbidities<br>(c) C9ORF72 and HIV characteristics
	APOE genotypes…………………………………………………….. (a) APOE genotypes, neurocognitive/functional assessments<br>(b) APOE genotype and comorbidities<br>(c) APOE genotype and HIV characteristics
	APOEε2………………………………………………………………… (a) APOEε2, neurocognitive/functional assessment<br>(b) APOEε2 and comorbidities<br>(c) APOEε2 and HIV characteristics
	APOEε3………………………………………………………………… (a) APOEε3, neurocognitive/functional assessment<br>(b) APOEε2 and comorbidities<br>(c) APOEε3 and HIV characteristics
	APOEε4………………………………………………………………… (a) APOEε4, neurocognitive/functional assessment<br>(b) APOEε4 and comorbidities<br>(c) APOEε4 and HIV characteristics
**Error! Reference source not found.**	H63D……………………………………………………………….… (a) H63D, neurocognitive/functional assessment<br>(b) H63D and comorbidities<br>(c) H63D and HIV characteristics
	Multiple Regressions………………………………………………… (a) MMSE score<br>(b) CDT score<br>(c) SPPB score

Table S1. Patients' characteristics (epidemiological, comorbidities, HIV)			
**Epidemiological**			
**Characteristics**	**Frequency (%)**		
	**Male (N=45)**	**Female (N=14)**	**Total (N=60)**
**Smoking**			
Yes	21 (50%)	1 (7,1%)	22 (39,3%)
No	21 (50%)	13 (92,9%)	34 (60,7%)
Total	42 (100%)	14 (100%)	56 (100%)
N-miss	3	1	4
**Falls during the last year**			
Yes	9 (22%)	6 (42,9%)	15 (27,3%)
No	32 (78%)	8 (57,1%)	40 (72,7%)
Total	41 (100%)	14 (100%)	55 (100%)
N-miss	4	1	5
	**Median (IQR)**		
**Age (years)**	68 (15)	71 (7,5)	69,5 (13,8)
**Years of schooling**	8 (6)	6,5 (6,8)	8 (8)
N-miss	6	1	7
**BMI**	24,4 (5)	25,2 (6,1)	24,7 (5)
**Years on ART**	18 (12)	18 (13)	18 (13)
**Prevalence of comorbidities for gender**			
**Characteristics**	**Frequency (%)**		**Frequency (%) (N=60)**
	**Male (N=45)**	**Female (N=15)**	
**Hypertriglyceridemia**			
No	28 (62,2%)	9 (60%)	37 (61,7%)
Yes	17 (37,8%)	6 (40%)	23 (38,3%)
**Diabetes**			
No	39 (86,7%)	11 (73,3%)	50 (83,3%)
Yes	6 (13,3%)	4 (26,7%)	10 (16,7%)
**Hypercholesterolaemia**			
No	30 (66,7%)	9 (60%)	39 (65%)
Yes	15 (33,3%)	6 (40%)	21 (35%)
**Cardiovascular disease**			
No	31 (68,9%)	13 (86,7%)	44 (73,3%)
Yes	14 (31,1%)	2 (13,3%)	16 (26,7%)
**Hypertension**			
No	31 (68,9%)	10 (66,7%)	41 (68,3%)
Yes	14 (31,1%)	5 (33,3%)	19 (31,7%)
**Liver disease**			
No	30 (66,7%)	10 (66,7%)	40 (66,7%)
Yes	15 (33,3%)	5 (33,3%)	20 (33,3%)
**Kidney disease**			
No	35 (77,8%)	12 (80%)	47 (78,3%)
Yes	10 (22,2%)	3 (20%)	13 (21,7%)
**Cancer**			
No	38 (84,4%)	12 (80%)	50 (83,3%)
Yes	7 (15,6%)	3 (20%)	10 (16,7%)
**Prevalence of multi-morbidity for gender**			
**Characteristics**	**Frequency (%)**		**Frequency (%) (N=60)**
	**Male (N=45)**	**Female (N=15)**	
**No comorbidities**	8 (17,8%)	1 (6,7%)	9 (15%)
**One comorbidity**	13 (28,9%)	3 (20%)	16 (26,7%)
**Comorbidities ≥ 2**	24 (53,3%)	11 (73,3%)	35 (58,3%)

Table S1. Patients' characteristics (epidemiological, comorbidities, HIV)			
**HIV characteristics**			
	**Median (IQR)**		
	**Male (N=45)**	**Female (N=15)**	**Total (N=60)**
**CD4⁺ T cells count**	542 (388)	589 (339)	546 (356,5)
**CD4%**	30 (14,3)	36,9 (14,7)	30 (16,2)
**Nadir CD4**	126 (226)	138 (183,5)	136 (224,5)
**Nadir CD4%**	13,1 (18,4)	16,6 (13,2)	14,2 (17,8)
N-Miss	5	0	5
**CD8⁺ T cells count**	719 (590)	561 (602)	672,5 (577,3)
**CD8%**	38 (17,4)	31,5 (15,5)	37 (17,5)
**Zenith CD8**	1542 (1069)	1344 (626)	1458,5 (886)
**Zenith CD8%**	54,9 (19,5)	48,6 (21,3)	54,6 (20)
	**Frequency (%)**		
**Previous virologic failure to ART (n°)**			
0	33 (73,3%)	11 (73,3%)	44 (73,3%)
1	7 (15,6%)	2 (13,3%)	9 (15%)
2	3 (6,7%)	0 (0%)	3 (5%)
3	1 (2,2%)	1 (6,7%)	2 (3,3%)
4	1 (2,2%)	0 (0%)	2 (3,3%)
**Plasmatic HIV RNA**			
Positive (> 20cp/mL)	4 (8,9%)	0 (0%)	4 (6,7%)
Negative (≤ 20 cp/mL)	41 (91,1%)	15 (100%)	56 (93,9%)
**PWH ART**			
ABC/3TC/DTG			13 (21,7%)
3TC/DTG			11 (18,3%)
TAF/FTC/BIC			8 (13,3%)
DTG/DRV/c			5 (8,3%)
FTC/TAF/RPV			4 (6,7%)
RPV/DTG			4 (6,7%)
No therapies			1 (1,7%)
Others*			14 (23,3%)

*Others include DOR/DTG; RAL/DRV/r; ABC/3TC/RAL; TAF/FTC/DTG; FTC/TAF/DRV/c; TAF/FTC/DOR; RAL/ETR; ABC/3TC/DRV/c

ABC, abacavir; BIC, bictegravir; c, cobicistat; DOR, doravirine; DTG, dolutegravir; DRV, darunavir; ETR, etravirine; FTC, emtricitabine; r, ritonavir; RAL, raltegravir; RPV, rilpivirine; TAF, tenofovir alafenamide; TDF, tenofovir disoproxil fumarate; 3TC, lamivudine

Table S2. Differences in neurocognitive /functional outcomes linked to comorbidities					
**Characteristics**	**Median (IQR)**	**Total (N=60)**	**Statistic**	**df**	**p-value**
	**MMSEscore**				
**Hypertriglyceridemia**			0,23	1	.630
No<br>Yes<br>N-Miss	26,3 (1,8)<br>26 (2,2)	35<br>19<br>6			
**Diabetes**			4,6	1	.031*
No<br>Yes<br>N-Miss	26,3 (2,1)<br>25,2 (2,3)	45<br>9<br>6			
**Hypercholesterolaemia**			0,3	1	.581
No<br>YesN-Miss<br>N-Miss	26,2 (1,1)<br>25,9 (2,4)	37<br>17<br>6			
**Cardiovascular disease**			0,24	1	.627
No<br>Yes<br>N-Miss	26,2 (2,5)<br>26,2 (2)	40<br>14<br>6			
**Hypertension**			0,1	1	.754
No<br>Yes<br>N-Miss	26,2 (3)<br>26,3 (1,8)	36<br>18<br>6			
**Liver disease**			0,1	1	.993
No<br>Yes<br>N-Miss	26,2 (2,6)<br>26,2 (1,5)	35<br>19<br>6			
**Kidney disease**			0,4	1	.510
No<br>Yes<br>N-Miss	26,3 (2,8)<br>25,9 (1,6)	44<br>10<br>6			
**Cancer**			0,5	1	.495
No<br>Yes<br>N-Miss	26,3 (2,9)<br>26 (2)	44<br>10<br>6			
	**CDT score**				
**Hypertriglyceridemia**			1,5	1	.226
No<br>Yes<br>N-Miss	8 (5)<br>10 (3)	35<br>19<br>6			
**Diabetes**			1,8	1	.178
No<br>Yes<br>N-Miss	9 (3)<br>5 (4)	45<br>9<br>6			
**Hypercholesterolaemia**			0,04	1	.840
No<br>Yes<br>N-Miss	9 (3)<br>8 (5)	37<br>17<br>6			
**Cardiovascular disease**			1,9	1	.168
No<br>Yes<br>N-Miss	8 (5)<br>9 (1,8)	40<br>14<br>6			
**Hypertension**			1,3	1	.258
No<br>Yes	9 (3,3)<br>7,5 (4,8)	36<br>18			

Table S2. Differences in neurocognitive /functional outcomes linked to comorbidities					
**Characteristics**	**Median (IQR)**	**Total (N=60)**	**Statistic**	**df**	**p-value**
N-Miss		6			
**Liver disease**			0,32	1	.574
No<br>Yes<br>N-Miss	8 (5)<br>9 (3)	35<br>19<br>6			
**Kidney disease**			0,1	1	.738
No<br>Yes<br>N-Miss	9 (5)<br>7,5 (4,8)	44<br>10<br>6			
**Cancer**			0,5	1	.468
No<br>Yes<br>N-Miss	9 (4,3)<br>8 (4,8)	44<br>10<br>6			
	**SPPB score**				
**Hypertriglyceridemia**			0,2	1	.656
No<br>Yes<br>N-Miss	11 (3)<br>10 (3)	35<br>20<br>5			
**Diabetes**			1,2	1	.283
No<br>Yes<br>N-Miss	11 (3)<br>9 (5)	46<br>9<br>5			
**Hypercholesterolaemia**			1,2	1	.272
No<br>Yes<br>N-Miss	11 (3)<br>10 (3,8)	37<br>18<br>5			
**Cardiovascular disease**			1,7	1	.189
No<br>Yes<br>N-Miss	11 (3)<br>10 (3)	40<br>15<br>5			
**Hypertension**			6,6	1	.010*
No<br>Yes<br>N-Miss	11,5 (2)<br>9 (3)	36<br>19<br>5			
**Liver disease**			1,3	1	.255
No<br>Yes<br>N-Miss	10 (3)<br>11 (2)	36<br>19<br>5			
**Kidney disease**			0,2	1	.626
No<br>Yes<br>N-Miss	10 (3)<br>11,5 (2,8)	45<br>10<br>5			
**Cancer**			0,6	1	.428
No<br>Yes<br>N-Miss	11 (3)<br>10,5 (4,3)	45<br>10<br>5			

Table S3. Spearman's Correlations				
**Variables**		**MMSE score**	**CDT score**	**SPPB score**
1. MMSE score	Spearman's rho	—		
	p-value	—		
	95% CI	—		
2. CDT score	Spearman's rho	0.312	—	
	p-value	0.021	—	
	95% CI	0.535 0.049	—	
3. SPPB score	Spearman's rho	0.033	0.041	—
	p-value	0.814	0.769	—
	95% CI	0.298 -0.237	0.305 -0.229	—
4. BMI	Spearman's rho	-0.098	-0.123	0.095
	p-value	0.481	0.376	0.490
	95% CI	0.174 -0.356	0.15 -0.378	0.351 -0.175
5. Years in ART	Spearman's rho	-0.045	0.263	-0.119
	p-value	0.746	0.055	0.386
	95% CI	0.225 -0.309	0.496 -0.005	0.151 -0.373
6. $CD4^{+}$ T cells count	Spearman's rho	0.114	0.136	0.262
	p-value	0.410	0.327	0.053
	95% CI	0.371 -0.158	0.39 -0.137	0.493 -0.003
7. CD4 %	Spearman's rho	0.104	-0.014	0.230
	p-value	0.454	0.919	0.092
	95% CI	0.362 -0.168	0.255 -0.281	0.466 -0.038
8. $CD8^{+}$ T cells count	Spearman's rho	0.034	0.072	0.057
	p-value	0.809	0.605	0.678
	95% CI	0.299 -0.236	0.333 -0.2	0.318 -0.211
9. CD8%	Spearman's rho	-0.053	-0.105	0.038
	p-value	0.706	0.449	0.785
	95% CI	0.218 -0.316	0.167 -0.363	0.3 -0.23
10. Nadir CD4	Spearman's rho	0.178	-0.093	0.165
	p-value	0.198	0.504	0.228
	95% CI	0.425 -0.094	0.179 -0.352	0.413 -0.105
11. Nadir CD4%	Spearman's rho	0.084	-0.110	0.076
	p-value	0.566	0.450	0.600
	95% CI	0.357 -0.202	0.176 -0.38	0.347 -0.207
12. Zenith CD8	Spearman's rho	-0.192	0.135	0.021
	p-value	0.164	0.331	0.876
	95% CI	0.08 -0.438	0.389 -0.138	0.285 -0.245
13. Zenith CD8%	Spearman's rho	-0.221	-0.078	0.173
	p-value	0.109	0.577	0.206
	95% CI	0.05 -0.461	0.194 -0.338	0.419 -0.096
14. Previous virologic failure to ART	Spearman's rho	0.037	0.202	-0.142
	p-value	0.788	0.143	0.303
	95% CI	0.302 -0.233	0.446 -0.069	0.129 -0.392
15. Age	Spearman's rho	-0.015	-0.27	-0.338
	p-value	0.915	0.049	0.012
	95% CI	0.254 -0.281	-0.002 -0.501	-0.08 -0.554

**Table S4. Prevalence of APOE genotype**				
**Characteristics**	**Frequency (%)**			**p-value**
	**Male (N=45)**	**Female (N=15)**	**Total (N=60)**	.004*
**APOEε3 ε3**	35 (77,8%)	7 (46,7%)	42 (70%)	
**APOEε3 ε4**	2 (4,4%)	6 (40%)	8 (13,3%)	
**APOEε2 ε4**	1 (2,2%)	1 (6,7%)	2 (3,3%)	
**APOEε2 ε3**	7 (15,6%)	1 (6,7%)	8 (13,3%)	
**APOEε3 ε2**	0 (0%)	0 (0%)	0 (0%)	
**APOEε4 ε4**	0 (0%)	0 (0%)	0 (0%)	
**APOEε2 ε2**	0 (0%)	0 (0%)	0 (0%)	
* p < .05				

Table S5. C9ORF72

Table S5a. C9ORF72, neurocognitive /functional assessment

**Tests**	**Alleles**	**Median (IQR)**	**Total (N=60)**	**Statistic**	**df**	**p-value**
	**C9ORF72**					
**MMSE score**				0,3	1	.584
	Expanded	26,1 (2,4)	4			
	Not Expanded	26,2 (2,6)	50			
N-Miss			6			
**CDT score**				0,019	1	.891
	Expanded	8,5 (1)	4			
	Not Expanded	9 (5)	50			
N-Miss			6			
**SPPB score**				0,7	1	.395
	Expanded	11 (3)	5			
	Not Expanded	9 (5)	50			
N-Miss			5			

Table S5b. C9ORF72 and the prevalence of comorbidities

**Characteristics**	**Frequency (%) (N=60)**		**Log Odds Ratio**	**95% CI**	**p-value**
	**C9ORF72**				
	Not expanded	Expanded			
**Hypertriglyceridemia**			1,13	-0,63 3,1	.240
No	34 (65,4%)	3 (37,5%)			
Yes	18 (34,6%)	5 (62,5%)			
Total	52 (100%)	8 (100%)			
**Diabetes**			0,59	-1,88 2,55	.610
No	44 (84,6%)	6 (75%)			
Yes	8 (13,4%)	2 (25%)			
Total	52 (100%)	8 (100%)			
**Hypercholesterolaemia**			1,3	-0,47 3,3	.114
No	36 (69,2%)	3 (37,5%)			
Yes	16 (30,8%)	5 (62,5%)			
Total	52 (100%)	8 (100%)			
**Cardiovascular disease**			1,2	-0,65 3	.192
No	40 (76,9%)	4 (50%)			
Yes	12 (23,1%)	4 (50%)			
Total	52 (100%)	8 (100%)			
**Hypertension**			-0,37	-2,8 1,5	1.000
No	35 (67,3%)	6 (75%)			
Yes	17 (32,7%)	2 (25%)			
Total	52 (1005)	8 (100%)			
**Liver disease**			-1,38	-5,3 0,8	.249
No	33 (63,5%)	7 (87,5%)			
Yes	19 (36,5%)	1 (12,5%)			
Total	52 (100%)	8 (100%)			
**Kidney disease**			-0,73	-4,6 1,5	.673
No	40 (76,9%)	7 (87,5%)			
Yes	12 (23,1%)	1 (12,5%)			
Total	52 (100%)	8 (100%)			
**Cancer**			-0,38	-4,3 1,9	1.000
No	43 (82,7%)	7 (87,5%)			
Yes	9 (17,3%)	1 (12,5%)			
Total	52 (100%)	8 (100%)			

Table S5. C9ORF72						
**Table S5c. C9ORF72 and HIV characteristics**						
**HIV values**	**Alleles**	**Median (IQR)**	**Total (N=60)**	**Statistic**	**df**	**p-value**
	**C9ORF72**					
**CD4^{+} T cell count**				4,6	1	.032*
	Expanded<br>Not expanded	776 (258,5)<br>513,5 (312)	8<br>52			
**CD4%**						.041*
	Expanded<br>Not expanded	38,6 (6,3)<br>28,5 (15,2)	52<br>8	4,2	1	
**Nadir CD4**				0,15	1	.695
	Expanded<br>Not expanded	156 (217,8)<br>136 (219,3)	8<br>52			
**Nadir CD4%**				0,07	1	.793
 <br>N-Miss	Expanded<br>Not expanded	13,2 (15,2)<br>14,2 (18,6)	8<br>47<br>5			
**CD8^{+} T cell count**				0,17	1	.679
	Expanded<br>Not expanded	530 (331,8)<br>700,5 (589,5)	8<br>52			
**CD8%**				3	1	.084
	Expanded<br>Not expanded	26,1 (6,1)<br>37,9 (15,5)	8<br>52			
**Zenith CD8**				0,27	1	.602
	Expanded<br>Not expanded	1235 (455,5)<br>1474,5 (1012)	8<br>52			
**Zenith CD8%**				0,9	1	.344
	Expanded<br>Not expanded	60,5 (17,9)<br>54 (20,1)	8<br>52			
* p < .05						

Table S6. APOE genotypes

Table S6a. APOE genotypes, neurocognitive /functional assessment

**test**	**APOE**	**Median (IQR)**	**Total (N=60)**	**Statistic**	**df**	**p-value**
**MMSE score**				2,2	3	.526
	**ε3 ε3**	26,2 (2,6)	38			
	**ε3 ε4**	27 (1,4)	7			
	**ε2 ε4**	22 (5)	2			
	**ε2 ε3**	26 (0,7)	7			
N-Miss			6			
**CDT score**				6,3	3	.097
	**ε3 ε3**	9 (3,8)	38			
	**ε3 ε4**	8 (3)	7			
	**ε2 ε4**	2,5 (1,5)	2			
	**ε2 ε3**	9 (3,5)	7			
N-Miss			6			
**SPPB score**				1,1	3	.772
	**ε3 ε3**	10 (3)	39			
	**ε3 ε4**	12 (2,5)	7			
	**ε2 ε4**	7 (5)	2			
	**ε2 ε3**	10 (5,5)	7			
N-Miss			5			

Table S6b. APOE genotype and comorbidites

**Characteristic**	**Frequency (%) (N=60)**				**Statistic**	**df**	**p-value**
	**APOE**						
**Hypertryglyceridemia**	**ε3 ε3**	**ε3 ε4**	**ε2 ε4**	**ε2 ε3**	2,5	3	.468
No	28 (6,7%)	5 (62,5%)	1 (50%)	3 (37,5%)			
Yes	14 (33,3%)	3 (37,5%)	1 (50%)	5 (62,5%)			
Total	42 (100%)	8 (100%)	8 (100%)	8 (100%)			
**Diabetes**					3,1	3	.379
No	37 (88,1%)	6 (75%)	1 (50%)	6 (75%)			
Yes	5 (11,9%)	2 (25%)	1 (50%)	2 (25%)			
Total	42 (100%)	8 (100%)	2 (100%)	8 (100%)			
**Hypercholesterolaemia**					0,29	3	.961
No	28 (66,7%)	5 (62,5%)	1 (50%)	5 (62,5%)			
Yes	14 (33,3%)	3 (37,5%)	1 (50%)	3 (37,5%)			
Total	42 (100%)	8 (100%)	2 (100%)	8 (100%)			
**Cardiovascular disease**					4,6	3	.203
No	28 (66,7%)	8 (100%)	2 (100%)	6 (45%)			
Yes	14 (33,3%)	0 (0%)	0 (0%)	2 (25%)			
Total	42 (100%)	8 (100%)	2 (100%)	8 (100%)			
**Hypertension**					1,2	3	.542
No	27 (64,3%)	7 (87,5%)	1 (50%)	6 (75%)			
Yes	15 (35,7%)	1 (12,5%)	1 (50%)	2 (25%)			
Total	42 (100%)	8 (100%)	2 (100%)	8 (100%)			
**Liver disease**					0,56	3	.905
No	28 (66,7%)	6 (75%)	1 (50%)	5 (62,5%)			
Yes	14 (33,3%)	2 (25%)	1 (50%)	3 (37,5%)			
Total	42 (100%)	8 (100%)	2 (100%)	8 (100%)			
**Kidney disease**					2,1	3	.454
No	33 (78,6%)	7 (87,5%)	2 (100%)	5 (62,5%)			
Yes	9 (21,4%)	1 (12,5%)	0 (0%)	3 (37,5%)			
Total	42 (100%)	8 (100%)	2 (100%)	8 (100%)			
**Cancer**					2,3	3	.518

Table S6. APOE genotypes							
No	36 (85,7%)	6 (75%)	1 (50%)	7 (87,5%)			
Yes	6 (14,3%)	2 (25%)	1 (50%)	1 (12,5%)			
Total	42 (100%)	8 (100%)	2 (100%)	8 (100%)			

Table S6c. APOE genotypes and HIV characteristics						
**HIV values**	**Alleles**	**Median (IQR)**	**Total (N=60)**	**Statistic**	**df**	**p-value**
	**APOE genotypes**					
**$CD4^+$ T cell count**				3,5	3	.325
	APOEε3 ε3<br>APOEε3 ε4<br>APOEε2 ε4<br>APOEε2 ε3	546 (332)<br>499 (345,3)<br>341,5 (14,5)<br>660,5 (357,5)	42<br>8<br>2<br>8			
**CD4%**				1,8	3	.604
	APOEε3 ε3<br>APOEε3 ε4<br>APOEε2 ε4<br>APOEε2 ε3	30 (15,1)<br>27,7 (12,6)<br>30 (12)<br>36,9 (11,3)	42<br>8<br>2<br>8			
**Nadir CD4**				1,3	3	.713
	APOEε3 ε3<br>APOEε3 ε4<br>APOEε2 ε4<br>APOEε2 ε3	151,5 (212,8)<br>86 (137,3)<br>242 (40)<br>152,5 (235,8)	42<br>8<br>2<br>8			
**Nadir CD4%**				3,9	3	.267
 <br><br><br>N-Miss	APOEε3 ε3<br>APOEε3 ε4<br>APOEε2 ε4<br>APOEε2 ε3	13,9 (17,7)<br>8,4 (8,5)<br>22,9 (6,3)<br>19,6 (12,5)	38<br>8<br>2<br><br>5			
**$CD8^+$ T cell count**				1	3	.809
	APOEε3 ε3<br>APOEε3 ε4<br>APOEε2 ε4<br>APOEε2 ε3	735 (591,2)<br>621,5 (556,5)<br>591 (275)<br>533 (275,5)	42<br>8<br>2<br>8			
**CD8%**				3,9	3	.271
	APOEε3 ε3<br>APOEε3 ε4<br>APOEε2 ε4<br>APOEε2 ε3	37,4 (18,1)<br>34,3 (20,9)<br>42,3 (2,3)<br>27,9 (10,4)	42<br>8<br>2<br>8			
**Zenith CD8**				9,1	3	.029*
	APOEε3 ε3<br>APOEε3 ε4<br>APOEε2 ε4<br>APOEε2 ε3	1576 (906)<br>1185,5 (597)<br>1130 (329)<br>906 (274,5)	42<br>8<br>2<br>8			
**Zenith CD8%**				1,8	3	.610
	APOEε3 ε3<br>APOEε3 ε4<br>APOEε2 ε4<br>APOEε2 ε3	55,1 (19,3)<br>54,6 (22,9)<br>53,5 (3,7)<br>45,9 (14,3)	42<br>8<br>2<br>8			

Table S7. APOEε2

Table S7a. APOEε2, neurocognitive /functional assessment

Tests	Alleles	Median (IQR)	Total (N=60)	Statistic	df	p-value
	**APOEε2**					
**MMSE score**				1,03	1	.311
	0	26,3 (2,7)	45			
	1	26 (1)	9			
N-Miss			6			
**CDT score**				0,67	1	.414
	0	9 (4)	45			
	1	8 (5)	9			
N-Miss			6			
**SPPB score**				0,7	1	.402
	0	11 (3)	46			
	1	10 (6)	9			
N-Miss			5			

Table S7b. APOEε2 and comorbidities

Characteristics	Frequency (%) (N=60)		Log Odds Ratio	95% CI	p-value
	**APOEε2**				
	0	1			
**Hypertriglyceridemia**			1,1	-0,5 2,8	.161
No	33 (66%)	4 (40%)			
Yes	17 (34%)	6 (60%)			
Total	50 (100%)	10 (100%)			
**Diabetes**			0,95	-1,1 2,7	.347
No	43 (86%)	7 (70%)			
Yes	7 (14%)	3 (30%)			
Total	50 (100%)	10 (1000%)			
**Hypercholesterolaemia**			0,25	-1,5 1,8	.729
No	33 (66%)	6 (60%)			
Yes	17 (34%)	4 (40%)			
Total	50 (100%)	10 (100%)			
**Cardiovascular disease**			-0,44	-2,8 1,3	.715
No	36 (72%)	8 (80%)			
Yes	14 (28%)	2 (20%)			
Total	50 (100%)	10 (100%)			
**Hypertension**			-0,092	-2 1,5	1.000
No	34 (68%)	7 (70%)			
Yes	16 (32%)	3 (30%)			
Total	50 (100%)	10 (100%)			
**Liver disease**			0,34	-1,4 1,9	.718
No	34 (68%)	6 (60%)			
Yes	16 (32%)	4 (40%)			
Total	50 (100%)	10 (100%)			
**Kidney disease**			0,53	-1,4 2,2	.675
No	40 (80%)	7 (70%)			
Yes	10 (20%)	3 (30%)			
Total	50 (100%)	10 (100%)			
**Cancer**			0,27	-2,2 2,1	.668
No	42 (84%)	8 (80%)			
Yes	8 (16%)	2 (20%)			
Total	50 (100%)	10 (100%)			

Table S7. APOEε2						
**Table S7c. APOEε2 and HIV characteristics**						
**HIV values**	**Alleles**	**Median (IQR)**	**Total (N=60)**	**Statistic**	**df**	**p-value**
	**APOEε2**					
**CD4^{+} T cell count**				0,01	1	.751
	0<br>1	546 (332,5)<br>599 (407,8)	50<br>10			
**CD4%**				1,4	1	.230
	0<br>1	29,4 (15,1)<br>36,9 (15,7)	50<br>10			
**Nadir CD4**				0,27	1	.606
	0<br>1	134 (213,3)<br>206,5 (212,5)	50<br>10			
**Nadir CD4%**				1,7	1	.187
 <br>N-Miss	0<br>1	12,6 (17,2)<br>19,6 (7,4)	46<br>9<br>5			
**CD8^{+} T cell count**				0,95	1	.331
	0<br>1	722,5 (593,3)<br>533 (446,3)	50<br>10			
**CD8%**				1,2	1	.271
	0<br>1	37,4 (18,4)<br>31,9 (13,1)	50<br>10			
**Zenith CD8**				6,6	1	.010*
	0<br>1	1521 (979,8)<br>906 (268)	50<br>10			
**Zenith CD8%**				1,3	1	.262
	0<br>1	55,1 (19,9)<br>48 (13,3)	50<br>10			
* p < .05						

Table S8. APOEε3

Table S8a. APOEε3, neurocognitive /functional assessment

Tests	Alleles	Median (IQR)	Total (N=60)	Statistic	df	p-value
	**APOEε3**					
**MMSE score**				0,611	1	.434
	No	22 (5)	2			
	Alleles ≥ 1	26,2 (2,3)	52			
N-Miss			6			
**CDT score**				5,5	1	.019*
	No	2,5 (2,1)	2			
	Alleles ≥ 1	8 (2,2)	52			
N-Miss			6			
**SPPB score**				0,196	1	.658
	No	7 (5)	2			
	Alleles ≥ 1	11 (3)	53			
N-Miss			5			
	**APOEε3**					
**MMSE score**				0,7	2	.705
	0	22 (5)	2			
	1	26,3 (1,1)	14			
	2	26,2 (2,6)	38			
N-Miss			6			
**CDT score**				5,8	2	.056
	0	2,5 (1,5)	2			
	1	8 (4,3)	14			
	2	9 (3,8)	38			
N-Miss			6			
**SPPB score**				0,2	1	.906
	0	7 (5)	2			
	1	11,5 (4,8)	14			
	2	10 (3)	39			
N-Miss			5			

Table S8b. APOEε3 and comorbidities

Characteristics	Frequency (%) (N=60)			Statistic	df	p-value
	APOEε3					
	0	1	2			
**Hypertriglyceridemia**				1,5	2	.477
No	1 (50%)	8 (50%)	28 (66,7%)			
Yes	1 (50%)	8 (50%)	14 (33,3%)			
Total	2 (100%)	16 (100%)	42 (100%)			
**Diabetes**				4,4	2	.361
No	1 (50%)	12 (75%)	37 (88,1%)			
Yes	1 (50%)	4 (25%)	5 (11,9%)			
Total	2 (100%)	16 (100%)	42 (100%)			
**Hypercholesterolaemia**				0,29	2	.864
No	1 (50%)	10 (62,5%)	28 (66,7%)			
Yes	1 (50%)	6 (37,5%)	14 (33,3%)			
Total	2 (100%)	16 (100%)	42 (100%)			
**Cardiovascular disease**				3,32	2	.190
No	2 (100%)	14 (87,5%)	28 (66,7%)			
Yes	0 (0%)	2 (12,5%)	14 (33,3%)			
Total	2 (100%)	16 (100%)	42 (100%)			

Table S8. APOEε3						
**Hypertension**				1,87	2	.394
No<br>Yes<br>Total	1 (50%)<br>1 (50%)<br>2 (100%)	13 (81,2%)<br>3 (18,8%)<br>16 (100%)	27 (64,3%)<br>15 (35,7%)<br>42 (100%)			
**Liver disease**				0,28	2	.869
No<br>Yes<br>Total	1 (50%)<br>1 (50%)<br>2 (100%)	11 (68,8%)<br>5 (31,2%)<br>16 (100%)	28 (66,7%)<br>14 (33,3%)<br>42 (100%)			
**Kidney disease**				0,66	2	.719
No<br>Yes<br>Total	2 (100%)<br>0 (0%)<br>2 (100%)	12 (75%)<br>4 (25%)<br>16 (100%)	33 (78,6%)<br>9 (21,4%)<br>42 (100%)			
**Cancer**				1,8	2	.402
No<br>Yes<br>Total	1 (50%)<br>1 (50%)<br>2 (100%)	13 (81,2%)<br>3 (18,8%)<br>16 (100%)	36 (85,7%)<br>6 (14,3%)<br>42 (100%)			

	**APOEε3**		**Log Odds Ratio**	**95% CI**	**p-value**
	No	Alleles ≥ 1			
**Hypertriglyceridemia**			-0,48	-4,9 3,9	1.000
No<br>Yes<br>Total	1 (50%)<br>1 (50%)<br>2 (100%)	36 (62,1%)<br>22 (37,9%)<br>58 (100%)			
**Diabetes**			1,65	-6,1 2,8	.308
No<br>Yes<br>Total	1 (50%)<br>1 (50%)<br>2 (100%)	49 (84,4%)<br>9 (15,5%)<br>58 (100%)			
**Hypercholesterolaemia**			-0,63	-5 3,8	1.000
No<br>Yes<br>Total	1 (50%)<br>1 (50%)<br>2 (100%)	38 (65,5%)<br>20 (34,5%)<br>58 (100%)			
**Cardiovascular disease**					-
No<br>Yes<br>Total	2 (100%)<br>0 (0%)<br>2 (100%)	42 (72,4%)<br>16 (27,6%)<br>58 (100%)			
**Hypertension**			-0,78	-5,2 3,6	.537
No<br>Yes<br>Total	1 (50%)<br>1 (50%)<br>2 (100%)	40 (69%)<br>18 (31%)<br>58 (100%)			
**Liver disease**			-0,71	-5,1 3,7	1.000
No<br>Yes<br>Total	1 (50%)<br>1 (50%)<br>2 (100%)	39 (67,2%)<br>19 (32,8%)<br>58 (100%)			
**Kidney disease**					-
No<br>Yes<br>Total	2 (100%)<br>0 (0%)<br>2 (100%)	45 (77,6%)<br>13 (22,4%)<br>58 (100%)			
**Cancer**			-1,7	-6,1 2,8	.308
No<br>Yes<br>Total	1 (50%)<br>1 (50%)<br>2 (100%)	49 (84,5%)<br>9 (15,5%)<br>58 (100%)			

Table S8c. APOEε3 and HIV characteristics

**HIV values**	**Alleles**	**Median (IQR)**	**Total (N=60)**	**Statistic**	**df**	**p-value**
	**APOEε3**					
**CD4^{+} T cell count**				2,6	2	.276

**Table S8. APOEε3**						
	0<br>1<br>2	341,5 (14,5)<br>590,5 (463,3)<br>546 (332)	2<br>16<br>42			
**CD4%**				0,83	2	.660
	0<br>1<br>2	30 (12)<br>33,7 (14,2)<br>30 (15,1)	2<br>16<br>42			
**Nadir CD4**				0,97	2	.615
	0<br>1<br>2	242 (40)<br>97,5 (244,5)<br>151,5 (212,8)	2<br>16<br>42			
**Nadir CD4%**				1,51	2	.471
 <br><br>N-Miss	0<br>1<br>2	22,9 (6,3)<br>11,6 (16,8)<br>13,9 (17,7)	2<br>15<br>38<br>5			
**$CD8^+$ T cell count**				0,53	2	.769
	0<br>1<br>2	591 (275)<br>549,5 (518,3)<br>735 (591,3)	2<br>16<br>42			
**CD8%**				1,55	2	.461
	0<br>1<br>2	42,3 (2,3)<br>29,8 (12,3)<br>37,4 (18,1)	2<br>16<br>42			
**Zenith CD8**				8,25	2	.016*
	0<br>1<br>2	1130 (329)<br>972,5 (505,5)<br>1576 (906)	2<br>16<br>42			
**Zenith CD8%**				0,13	2	.939
	0<br>1<br>2	53,5 (3,7)<br>48,8 (24,7)<br>55,1 (19,3)	2<br>16<br>42			
* p < .05						

Table S9. APOEε4

Table S9a. APOEε4, neurocognitive /functional assessment

Tests	Alleles	Median (IQR)	Total (N=60)	Statistic	df	p-value
	**APOEε4**					
**MMSE score**				0,46	1	.499
	0	26,2 (2,4)	45			
	1	27 (1)	9			
N-Miss			6			
**CDT score**				3,3	1	.068
	0	9 (4)	45			
	1	8 (4)	9			
N-Miss			6			
**SPPB score**				0,2	1	.637
	0	10 (3)	46			
	1	12 (4)	9			
N-Miss			5			

Table S9b. APOEε4 and comorbidities

Characteristics	Frequency (%) (N=60)		Log Odds Ratio	95% CI	p-value
	**APOEε4**				
	0	1			
**Hypertriglyceridemia**			0,08	-1,6 1,7	1.000
No	31 (62%)	6 (60%)			
Yes	19 (38%)	4 (40%)			
Total	50 (100%)	10 (100%)			
**Diabetes**			0,95	-1,1 2,7	.347
No	43 (86%)	7 (70%)			
Yes	7 (14%)	3 (30%)			
Total	50 (100%)	10 (100%)			
**Hypercholesterolaemia**			0,25	-1,5 1,8	.729
No	33 (66%)	6 (60%)			
Yes	17 (34%)	4 (40%)			
Total	50 (100%)	10 (100%)			
**Cardiovascular disease**					-
No	34 (68%)	10 (100%)			
Yes	16 (32%)	0 (0%)			
Total	50 (100%)	10 (100%)			
**Hypertension**			-0,7	-3,1 1,1	.480
No	33 (66%)	8 (80%)			
Yes	17 (34%)	2 (20%)			
Total	50 (100%)	10 (100%)			
**Liver disease**			-0,12	-2,1 1,5	1.000
No	33 (66%)	7 (70%)			
Yes	17 (34%)	3 (30%)			
Total	50 (100%)	10 (100%)			
**Kidney disease**			-1,03	-4,9 1,1	.436
No	38 (76%)	9 (90%)			
Yes	12 (24%)	1 (10%)			
Total	50	10 (100%)			
**Cancer**			0,95	-1,1 2,7	.347
No	43 (86%)	7 (70%)			

Table S9. APOEε4					
Yes	7 (14%)	3 (30%)			
Total	50 (100%)	10 (100%)			

Table S9. APOEε4						
**Table S9c. APOEε4 and HIV characteristics**						
**HIV values**	**Alleles**	**Median (IQR)**	**Total (N=60)**	**Statistic**	**df**	**p-value**
	**APOEε4**					
**CD4^{+} T cell count**				0,98	1	.321
	0	552 (374)	50			
	1	405 (257)	10			
**CD4%**				0,04	1	.843
	0	30,8 (15,5)	50			
	1	27,7 (17,6)	10			
**Nadir CD4**				0,13	1	.721
	0	151,5 (221)	50			
	1	119,5 (209,8)	10			
**Nadir CD4%**				0,8	1	.371
	0	14,8 (19,1)	45			
	1	10,7 (11,1)	10			
N-Miss			5			
**CD8^{+} T cell count**				0,002	1	.960
	0	691 (573,3)	50			
	1	621,5 (537,3)	10			
**CD8%**				1,1	1	.302
	0	36,7 (16,7)	50			
	1	39,3 (19)	10			
**Zenith CD8**				1,77	1	.184
	0	1488,5 (1004,5)	50			
	1	1185,5 (626,3)	10			
**Zenith CD8%**				0,47	1	.494
	0	54,6 (20,4)	50			
	1	53,5 (18,5)	10			

Table S10. H63D

Table S10a. H63D, neurocognitive /functional assessment

**Tests**	**Alleles**	**Median (IQR)**	**Total (N=60)**	**Statistic**	**df**	**p-value**
	**HFE H63D**					
**MMSE score**				0,9	1	.346
	0	26,3 (2,9)	38			
	1	26 (1,7)	16			
N-Miss			6			
**CDT score**				0,03	1	.875
	0	9 (5)	38			
	1	8,5 (4,3)	16			
N-Miss			6			
**SPPB score**				1,9	1	.165
	0	11 (3)	38			
	1	10 (5)	17			
N-Miss			5			

Table S10b. H63D and comorbidities

**Characteristics**	**Frequency (%) (N=60)**		**Log Odds Ratio**	**95% CI**	**p-value**
	**HFE H63D D**				
	0	1			
**Hypertriglyceridemia**			1,34	0,06 2,7	.023*
No	30 (71,4%)	7 (38,9%)			
Yes	12 (28,6%)	11 (61,1%)			
Total	42 (100%)	18 (100%)			
**Diabetes**			0,53	-1,2 2,1	.468
No	36 (85,7%)	14 (77,8%)			
Yes	6 (14,3%)	4 (22,2%)			
Total	42 (100%)	18 (100%)			
**Hypercholesterolaemia**			0,24	-1,1 1,5	.771
No	28 (66,7%)	11 (61,1%)			
Yes	14 (33,3%)	7 (38,9%)			
Total	42 (100%)	18 (100%)			
**Cardiovascular disease**			0,079	-1,4 1,5	1.000
No	31 (73,8%)	13 (72,2%)			
Yes	11 (26,2%)	5 (27,8%)			
Total	42 (100%)	18 (100%)			
**Hypertension**			0,46	-0,9 1,8	.547
No	30 (71,4%)	11 (61,1%)			
Yes	12 (28,6%)	7 (38,9%)			
Total	42 (100%)	18 (100%)			
**Liver disease**			1,02	-0,3 2,3	.134
No	31 (73,8%)	9 (50%)			
Yes	11 (26,2%)	9 (50%)			
Total	42 (100%)	18 (100%)			
**Kidney disease**			0,9	-0,6 2,4	.181
No	35 (83,3%)	12 (66,7%)			
Yes	7 (16,7%)	6 (33,3%)			
Total	42 (100%)	18 (100%)			
**Cancer**			1,03	-0,6 2,7	.149
No	37 (88,1%)	13 (72,2%)			
Yes	5 (11,9%)	5 (27,8%)			
Total	42 (100%)	18 (100%)			

Table S10. H63D						
**Table S10c. H63D and HIV characteristics**						
**HIV values**	**Alleles**	**Median (IQR)**	**Total (N=60)**	**Statistic**	**df**	**p-value**
	**HFE H63D**					
**CD4⁺ T cell count**				0,38	1	.540
	0<br>1	530 (431,8)<br>592 (281,5)	42<br>18			
**CD4%**				0,52	1	.473
	0<br>1	31 (17,6)<br>27,7 (11,1)	42<br>18			
**Nadir CD4**				1,31	1	.252
	0<br>1	206 (235,3)<br>100,5 (139,3)	42<br>18			
**Nadir CD4%**				0,48	1	.489
 <br>N-Miss	0<br>1	16,7 (15,5)<br>11,6 (16,3)	38<br>17<br>5			
**CD8⁺ T cell count**				2,7	1	.098
	0<br>1	558,5 (470)<br>958,5 (641,8)	42<br>18			
**CD8%**				0,89	1	.345
	0<br>1	37 (16)<br>37 (20)	42<br>18			
**Zenith CD8**				0,02	1	.897
	0<br>1	1452 (912)<br>1531 (789)	42<br>18			
**Zenith CD8%**				3	1	.084
	0<br>1	57,7 (19)<br>46,1 (15,9)	42<br>18			
* p < .05<br>Note: the only patient with the double H63D allele was included in the group of patients that were heterozygous for the variant.						

Multiple regressions

Table S11a. Multiple regressions of MMSE score

Model Summary – MMSE score

Model	R	R²	Adjusted R²	RMSE
H_0	0.000	0.000	0.000	0.082
H_1	0.531	0.282	-0.263	0.093

ANOVA

Model		Sum of Squares	df	Mean Square	F	p
H_1	Regression	0.097	22	0.004	0.517	0.943
	Residual	0.249	29	0.009		
	Total	0.346	51			

Note. The intercept model is omitted, as no meaningful information can be shown. Variables of the model were introduced using the enter method.

Coefficients

							95% CI		Collinearity Statistics	
Model		Unstandardized	Standard Error	Standardized	t	p	Lower	Upper	Tolerance	VIF
H_0	(Intercept)	1.406	0.011		123.06	< .001	1.383	1.429		
H_1	(Intercept)	1.284	0.410		3.133	0.004	0.446	2.123		
	C9ORF72 (alleles ≥ 9)	-0.023	0.068	-0.076	-0.345	0.732	-0.162	0.115	0.505	1.981
	APOEε2	0.033	0.054	0.145	0.614	0.544	-0.077	0.142	0.442	2.264
	APOEε3	0.185	0.113	0.436	1.631	0.114	-0.047	0.417	0.346	2.887
	APOEε4	0.078	0.059	0.362	1.330	0.194	-0.042	0.198	0.335	2.987
	HFE H63D	-0.055	0.039	-0.307	-1.416	0.168	-0.135	0.025	0.527	1.898
	$CD4^+$ T cells count	-0.033	0.112	-0.099	-0.295	0.770	-0.262	0.196	0.219	4.562
	NadirCD4	0.029	0.045	0.183	0.648	0.522	-0.063	0.121	0.310	3.230
	$CD8^+$ T cells count	0.078	0.103	0.262	0.755	0.456	-0.132	0.288	0.207	4.838
	Zenith CD8	-0.107	0.104	-0.317	-1.027	0.313	-0.319	0.106	0.260	3.850
	Age	0.002	0.002	0.192	0.632	0.532	-0.003	0.007	0.270	3.703
	Years on ART	0.002	0.002	0.210	0.947	0.352	-0.002	0.007	0.505	1.978
	Previous virologic failure to ART	-0.001	0.018	-0.016	-0.070	0.944	-0.039	0.036	0.460	2.172
	Plasmatic HIV RNA	0.021	0.062	0.070	0.346	0.732	-0.105	0.148	0.608	1.644
	BMI	-0.002	0.003	-0.203	-0.780	0.442	-0.009	0.004	0.365	2.737
	Hypertriglyceridemia	0.055	0.056	0.321	0.988	0.331	-0.059	0.169	0.235	4.263
	Diabetes	-0.035	0.056	-0.153	-0.620	0.540	-0.148	0.079	0.409	2.447
	Hypercholesterolaemia	-0.063	0.067	-0.363	-0.939	0.356	-0.201	0.074	0.166	6.038
	Cardiovascular disease	0.006	0.036	0.034	0.171	0.865	-0.068	0.081	0.635	1.575
	Hypertension	0.037	0.044	0.210	0.834	0.411	-0.053	0.126	0.390	2.562
	Liver disease	-0.020	0.038	-0.121	-0.537	0.596	-0.099	0.058	0.488	2.048
	Kidney disease	0.032	0.051	0.148	0.621	0.539	-0.073	0.137	0.439	2.279
	Cancer	0.005	0.044	0.023	0.107	0.915	-0.085	0.094	0.552	1.811

Casewise Diagnostics

Case Number	Std. Residual	MMSE	Predicted Value	Residual	Cook's Distance
38	-4.726	0.950	1.320	-0.370	0.386

Table S11b. Multiple regressions of CDT score

Model Summary - CDT score

Model	R	R^2	Adjusted R^2	RMSE
H_0	0.000	0.000	0.000	0.186
H_1	0.720	0.519	0.177	0.169

ANOVA

Model		Sum of Squares	df	Mean Square	F	p
H_1	Regression	0.952	22	0.043	1.517	0.140
	Residual	0.884	31	0.029		
	Total	1.836	53			

Note. The intercept model is omitted, as no meaningful information can be shown. Variables of the model were introduced using the enter method.

Coefficients

Model		Unstandardized	Standard Error	Standardized	t	p	95% CI Lower	95% CI Upper	Collinearity Statistics Tolerance	Collinearity Statistics VIF
H_0	(Intercept)	0.861	0.025		33.982	< .001	0.810	0.912		
H_1	(Intercept)	0.531	0.737		0.721	0.477	-0.972	2.035		
	C9ORF72 (alleles ≥ 9)	0.076	0.120	0.108	0.632	0.532	-0.169	0.321	0.532	1.879
	APOEε2	-0.037	0.093	-0.074	-0.391	0.699	-0.227	0.154	0.435	2.299
	APOEε3	0.435	0.199	0.445	2.182	0.037	0.028	0.841	0.373	2.679
	APOEε4	-0.018	0.106	-0.037	-0.173	0.863	-0.236	0.199	0.336	2.980
	HFE H63D	-0.045	0.071	-0.111	-0.631	0.533	-0.189	0.100	0.505	1.982
	$CD4^+$ T cells count	0.111	0.190	0.146	0.586	0.562	-0.277	0.499	0.251	3.986
	Nadir CD4	-0.054	0.075	-0.151	-0.713	0.481	-0.208	0.100	0.346	2.893
	$CD8^+$ T cells count	-0.077	0.169	-0.113	-0.454	0.653	-0.423	0.269	0.251	3.983
	Zenith CD8	0.076	0.178	0.099	0.426	0.673	-0.287	0.439	0.290	3.445
	Age	-0.005	0.004	-0.289	-1.297	0.204	-0.014	0.003	0.313	3.194
	Years on ART	0.002	0.004	0.083	0.481	0.634	-0.006	0.010	0.521	1.919
	Previous virologic failure to ART	0.005	0.032	0.030	0.170	0.866	-0.060	0.071	0.500	1.999
	Plasmatic HIV RNA	0.025	0.111	0.035	0.224	0.824	-0.202	0.251	0.625	1.599
	BMI	-0.001	0.006	-0.045	-0.214	0.832	-0.013	0.010	0.353	2.833
	Hypertriglyceridemia	0.111	0.094	0.288	1.191	0.243	-0.079	0.302	0.265	3.776
	Diabetes	-0.008	0.093	-0.017	-0.091	0.928	-0.198	0.181	0.442	2.261
	Hypercholesterolaemia	-0.014	0.098	-0.035	-0.140	0.890	-0.214	0.187	0.254	3.943
	Cardiovascular disease	0.051	0.065	0.122	0.784	0.439	-0.082	0.185	0.644	1.554
	Hypertension	0.039	0.078	0.100	0.499	0.621	-0.120	0.198	0.391	2.558
	Liver disease	-0.015	0.068	-0.038	-0.216	0.830	-0.152	0.123	0.507	1.971
	Kidney disease	0.009	0.090	0.018	0.095	0.925	-0.176	0.193	0.430	2.328
	Cancer	-0.012	0.076	-0.026	-0.164	0.871	-0.167	0.142	0.610	1.640

Casewise Diagnostics

Case Number	Std. Residual	CDT	Predicted Value	Residual	Cook's Distance
.	.	.	.	.	.

Table S11c. Multiple regressions of SPPB score

Model Summary – SPPB score

Model	R	R^2	Adjusted R^2	RMSE
H_0	0.000	0.000	0.000	0.129
H_1	0.663	0.440	0.042	0.126

ANOVA

Model		Sum of Squares	df	Mean Square	F	p
H_1	Regression	0.386	22	0.018	1.106	0.391
	Residual	0.492	31	0.016		
	Total	0.879	53			

Note. The intercept model is omitted, as no meaningful information can be shown. Variables of the model were introduced using the enter method.

Coefficients

							95% CI		Collinearity Statistics	
Model		Unstandardized	Standard Error	Standardized	t	p	Lower	Upper	Tolerance	VIF
H_0	(Intercept)	0.992	0.018		56.609	< .001	0.957	1.027		
H_1	(Intercept)	0.936	0.550		1.701	0.099	-0.186	2.058		
	C9ORF72 (alleles ≥ 9)	-0.023	0.090	-0.048	-0.259	0.798	-0.206	0.160	0.532	1.879
	APOEε2	-0.095	0.070	-0.279	-1.369	0.181	-0.238	0.047	0.435	2.299
	APOEε3	0.222	0.149	0.329	1.494	0.145	-0.081	0.525	0.373	2.679
	APOEε4	-0.041	0.079	-0.121	-0.522	0.605	-0.203	0.121	0.336	2.980
	HFE H63D	-0.007	0.053	-0.024	-0.125	0.901	-0.114	0.101	0.505	1.982
	$CD4^+$ T cells count	0.017	0.142	0.033	0.122	0.903	-0.272	0.307	0.251	3.986
	Nadir CD4	0.044	0.056	0.178	0.777	0.443	-0.071	0.159	0.346	2.893
	$CD8^+$ T cells count	-0.041	0.126	-0.088	-0.327	0.746	-0.299	0.217	0.251	3.983
	Zenith CD8	0.014	0.133	0.026	0.103	0.919	-0.257	0.284	0.290	3.445
	Age	-0.004	0.003	-0.294	-1.224	0.230	-0.010	0.003	0.313	3.194
	Years on ART	-0.001	0.003	-0.086	-0.460	0.648	-0.007	0.005	0.521	1.919
	Previous virologic failure to ART	-0.006	0.024	-0.050	-0.261	0.796	-0.055	0.043	0.500	1.999
	Plasmatic HIV RNA	-0.018	0.083	-0.036	-0.212	0.833	-0.186	0.151	0.625	1.599
	BMI	0.003	0.004	0.172	0.758	0.454	-0.005	0.012	0.353	2.833
	Hypertriglyceridemia	0.036	0.070	0.134	0.511	0.613	-0.107	0.178	0.265	3.776
	Diabetes	0.043	0.069	0.125	0.618	0.541	-0.098	0.184	0.442	2.261
	Hypercholesterolaemia	0.031	0.073	0.114	0.429	0.671	-0.118	0.181	0.254	3.943
	Cardiovascular disease	-0.018	0.049	-0.062	-0.368	0.715	-0.117	0.082	0.644	1.554
	Hypertension	-0.043	0.058	-0.157	-0.731	0.471	-0.161	0.076	0.391	2.558
	Liver disease	0.011	0.050	0.042	0.222	0.825	-0.092	0.114	0.507	1.971
	Kidney disease	-0.013	0.067	-0.039	-0.193	0.849	-0.150	0.124	0.430	2.328
	Cancer	-0.044	0.057	-0.134	-0.780	0.442	-0.159	0.071	0.610	1.640

Casewise Diagnostics

Case Number	Std. Residual	SPPB	Predicted Value	Residual	Cook's Distance
26	4.253	1.080	0.767	0.313	1.523
32	-4.253	0.300	0.613	-0.313	1.523
